# Optimizing mechanical properties of Fe_26.7_Co_26.7_Ni_26.7_Si_8.9_B_11_ high entropy alloy by inducing hypoeutectic to quasi-duplex microstructural transition

**DOI:** 10.1038/s41598-018-36464-3

**Published:** 2019-01-23

**Authors:** Ze-Qun Zhang, Kai-Kai Song, Shu Guo, Qi-Sen Xue, Hui Xing, Chong-De Cao, Fu-Ping Dai, Bernhard Völker, Anton Hohenwarter, Tapabrata Maity, Niraj Chawake, Jeong-Tae Kim, Li Wang, Ivan Kaban, Jürgen Eckert

**Affiliations:** 1 School of Mechanical, Electrical & Information Engineering, Shandong University (Weihai), 264209 Weihai, China; 2 School of Materials Science and Engineering, Harbin Institute of Technology, 150001 Harbin, China; 3 Department of Physics, School of Science, Northwestern Polytechnical University, 710072 Xi’an, China; 4 Erich Schmid Institute of Materials Science, Austrian Academy of Sciences, A-8700 Leoben, Austria; 5 Materials Chemistry, RWTH-Aachen, D-52074 Aachen, Germany; 6 Max-Plank-Institut für Eisenforschung GmbH, D-40237 Düsseldorf, Germany; 7 Department Materials Physics, Montanuniversität Leoben, A-8700 Leoben, Austria; 8 IFW Dresden, Institute for Complex Materials, D-01069 Dresden, Germany

## Abstract

High-entropy alloys (HEAs) have inspired considerable interest due to their attractive physical and mechanical properties. In this work, the microstructural evolution induced by different heat treatments on rapidly solidified hypoeutectic precursors of a Fe_26.7_Co_26.7_Ni_26.7_Si_8.9_B_11_ HEA is investigated and correlated with the corresponding mechanical properties. The microstructures of the rapidly solidified precursors are composed of primary *fcc* solid solution dendrites embedded in a eutectic matrix. When the samples are annealed at different temperatures after furnace cooling or quenching, respectively, the eutectic structure gradually decomposes into *fcc*, tetragonal (Fe,Co)_2_B, and hexagonal Ni_31_Si_12_ crystals with increasing annealing temperature, leading to a gradual increase of the content of the *fcc* crystals and both their aggregation and coarsening. Then the dominant structural framework gradually transforms from eutectic structures to *fcc* dendrites and ultimately the (Fe,Co)_2_B crystals become isolated as dominant reinforcement particles distributed in the interdendritic regions. This gradual microstructural transition from hypoeutectic to quasi-duplex structures leads to the change of the dominant deformation mechanism from crack-controlled to dislocation-dominated deformation, which allows to control both ductility and strength in a wide range. Hence, this study provides some guideline for how to tune the microstructure and mechanical properties of HEAs.

## Introduction

In recent years, high entropy alloys (HEAs) with superior mechanical properties have been designed in attempt to overcome the trade-off between strength and ductility in many classes of materials^1–6^. Besides, their attractive physical and mechanical properties such as high hardness, good wear resistance, good tribological properties, high resistance against softening at elevated temperatures, and favorable corrosion resistance make HEAs potential candidates for high-temperature applications^1–8^. Since HEAs typically contain multiple principle elements in equal or near equal atomic ratios ranging from 5 at.% to 35 at.%, it is easy to obtain simple and disordered solid-solutions, such as face-centered-cubic (*fcc*) or body-centered cubic (*bcc*) structures, or mixtures of them due to the high configurational entropy of alloys^1–7,9–12^. Even a hexagonal close-packed structured (*hcp*) phase can be induced in a HEA matrix in some cases^1–7,9–11,13,14^. These structural characteristics are ascribed to the sluggish diffusion of constituents, the so-called “cocktail effect”, and the large lattice distortion in such multiple-element mixtures^1–7,11,15–17^. As a result, the configurational entropy of these multi-component solid solutions can overcome the enthalpy of formation of competing intermetallic phases^18,19^. Until now, different HEA alloy systems have been developed from different base alloys, such as Fe-Mn-Co-Cr, Fe-Co-Ni-Cr-Mn/Al, Ta-Nb-Hf-Zr-Ti, Nb-Mo-Ta-W, V-Nb-Mo-Ta-W, Mo-Nb-Ta-W, and Hf-Nb-Ti-Zr alloys etc.^20–31^. Furthermore, it has been shown that the mechanical properties of HEAS are strongly affected by their often coarse, heterogeneous grain structures and chemical gradients (segregation) in the microstructures as a result of dendritic solidification during casting^20–32^. Therefore, micro-alloying additions, heat treatments, cold working, or/and other methods have been adopted to tailor the microstructures and properties of HEAs^5,20–33^, which needs deep and systematic investigations.

On the other hand, most of constituents in multicomponent HEAs usually possess relatively large atomic weights, resulting in a larger apparent density and thus a lower specific strength than conventional steels, titanium alloys, and aluminum alloys^34^. In order to increase the specific strength of HEAs, the element Al is usually adopted during alloy design^1–6,8^. Recent studies have shown that when B, Si, P, or/and C with relatively small atomic weights are introduced into Fe-Co-Ni medium entropy alloys^35–38^, high entropy metallic glasses and glass-matrix composites containing nanoscale *fcc* solid solution crystals can be achieved^39,40^, which also exhibit relatively good soft magnetic properties. It was found that a solid state phase transition from *fcc* to *bcc* solid solution can be induced by annealing such high entropy metallic glass composites^39,40^. However, this phenomenon is still far from being well understood. Moreover, the microstructures and mechanical properties of the crystalline counterparts of such high entropy metallic glasses and their composites are rarely investigated. In this work, Fe_26.7_Co_26.7_Ni_26.7_Si_8.9_B_11_ high-entropy hypoeutectic precursors were fabricated by rapid solidification and different heat treatments were performed to adjust their microstructural features in order to improve their room-temperature mechanical properties. The corresponding deformation mechanisms were also analyzed.

## Results and Discussion

### Microstructural features of as-cast and annealed HEA samples

**Figure 1:**
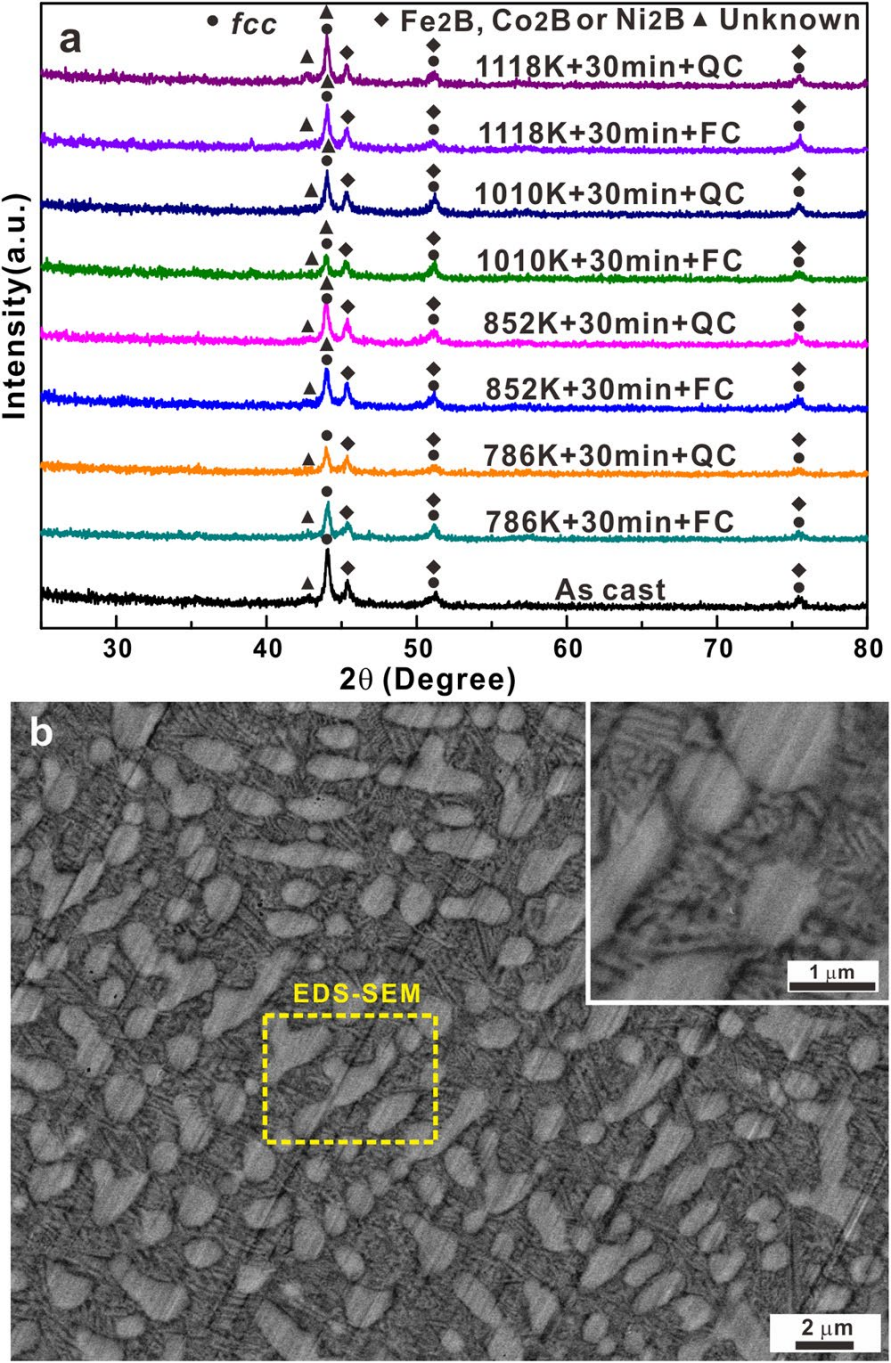
(**a**) XRD patterns of the as-cast alloy and samples annealed at different temperatures followed by furnace cooling (FC) or quenching (QC), and (**b**) SEM image of an as-cast sample.

**Figure 2:**
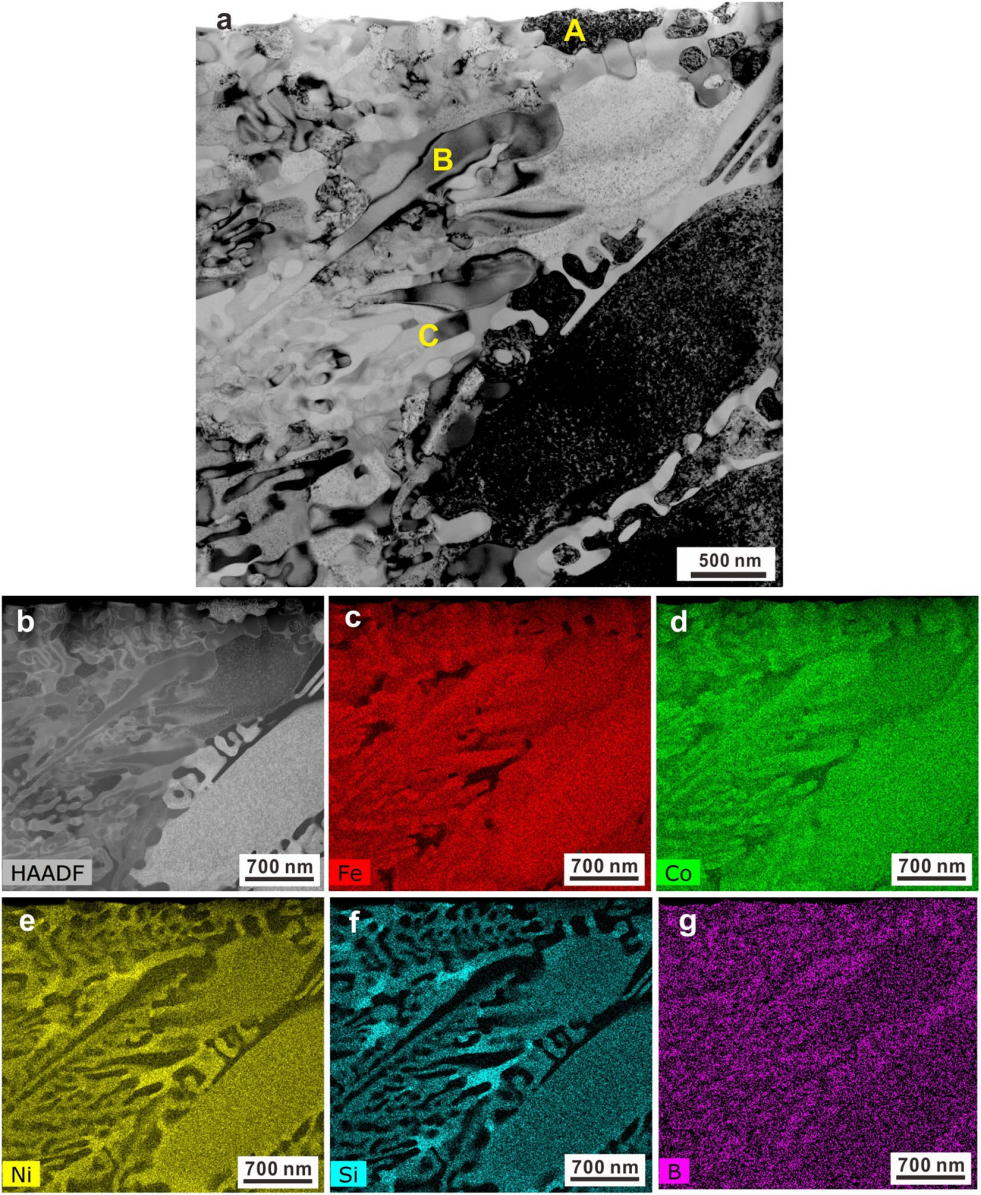
(**a**) Bright-field TEM image and (**b**) HAADF-STEM image of an as-cast sample as well as its corresponding chemical distributions of elements (**c**) Fe, (**d**) Co, (**e**) Ni, (**f**) Si, and (**g**) B.

**Figure 3:**
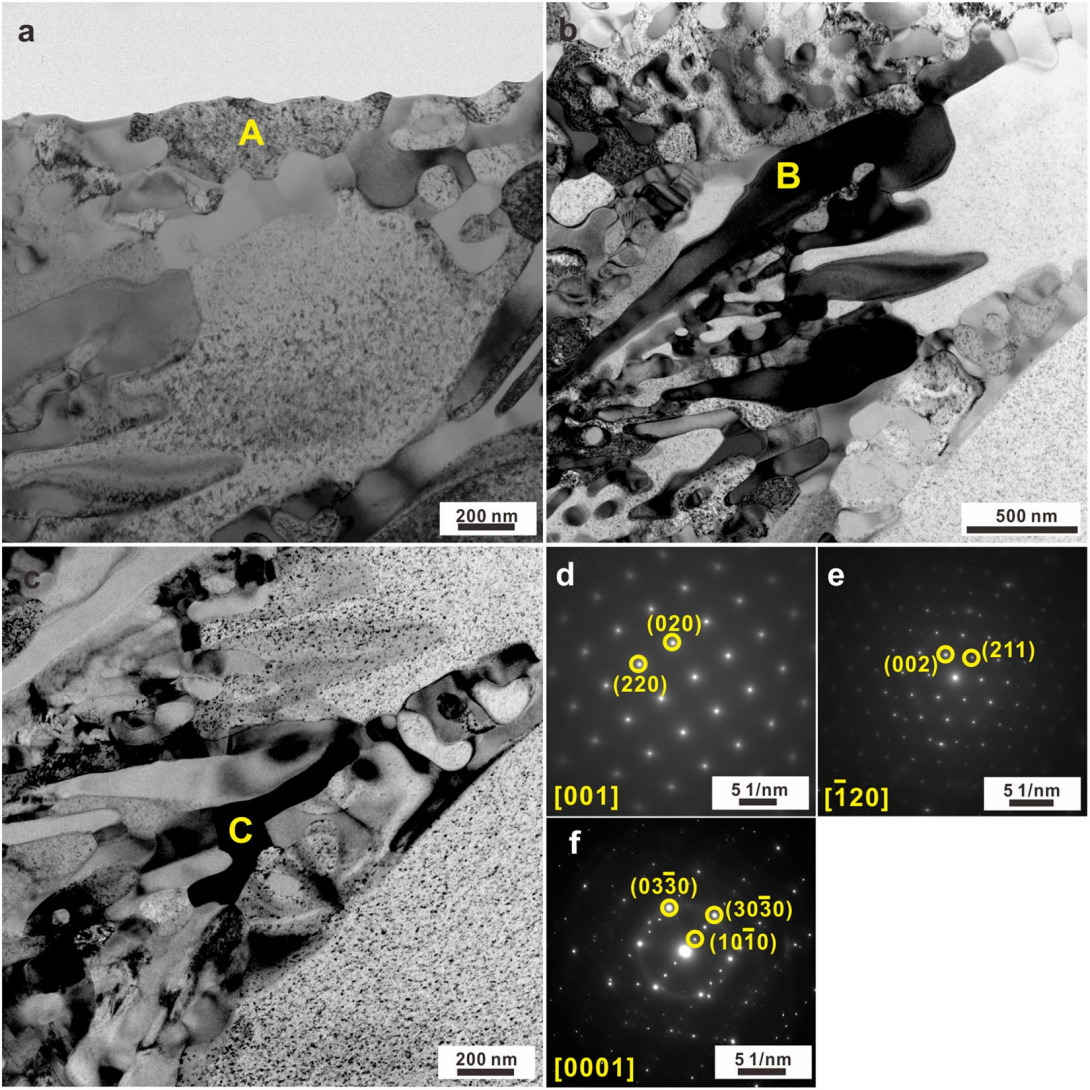
Bright-field TEM images of the regions (**a**) A, (**b**) B, and (**c**) C in an as-cast sample, and their corresponding SAED patterns (**d**–**f**).

**Figure 4:**
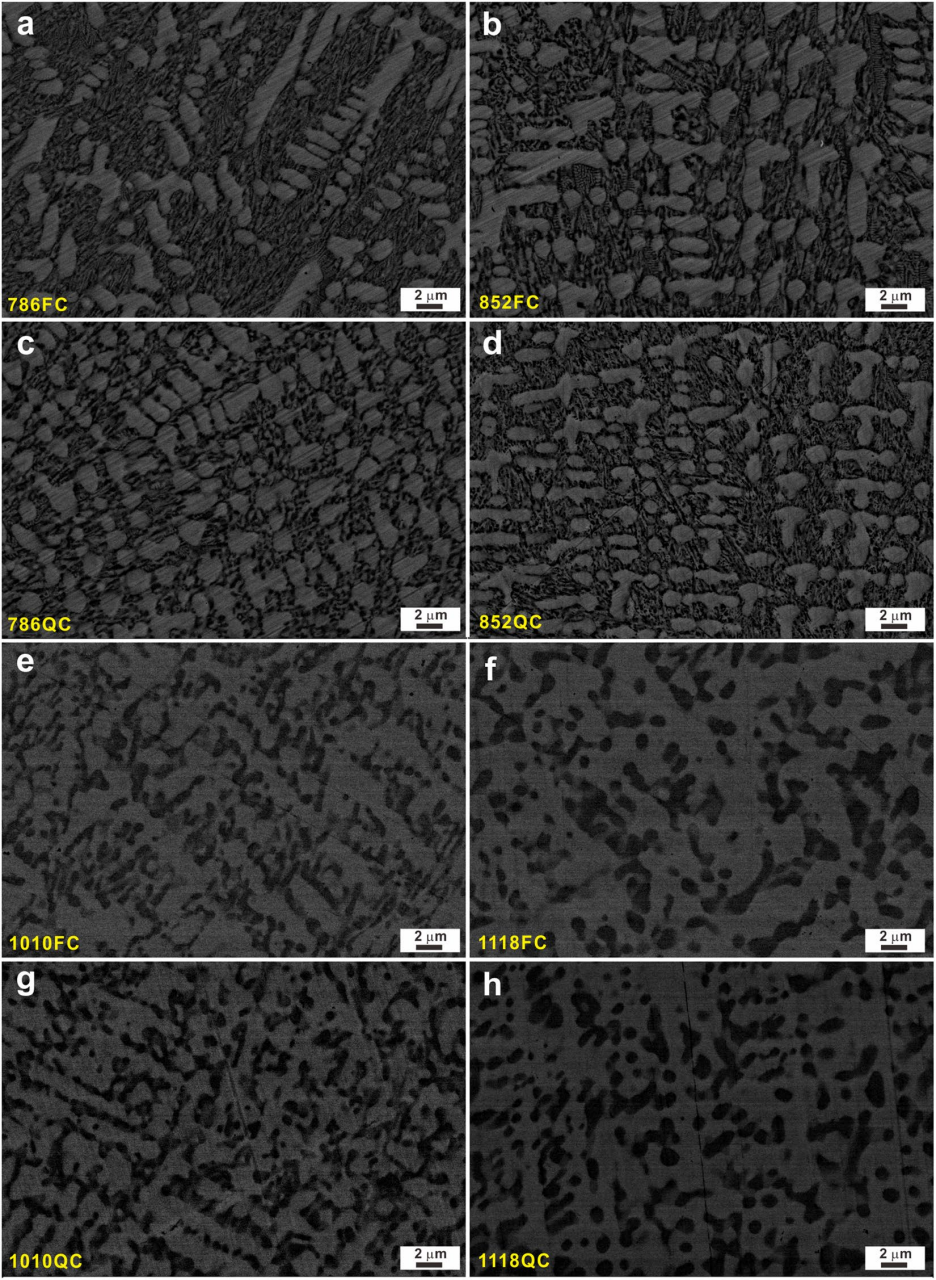
SEM images of the (**a**) 786FC, (**b**) 852FC, (**c**) 786QC, (**d**) 852QC, (**e**) 1010FC, (**f**) 1118FC, (**g**) 1010QC, and (**h**) 1118QC samples.

**Figure 5:**
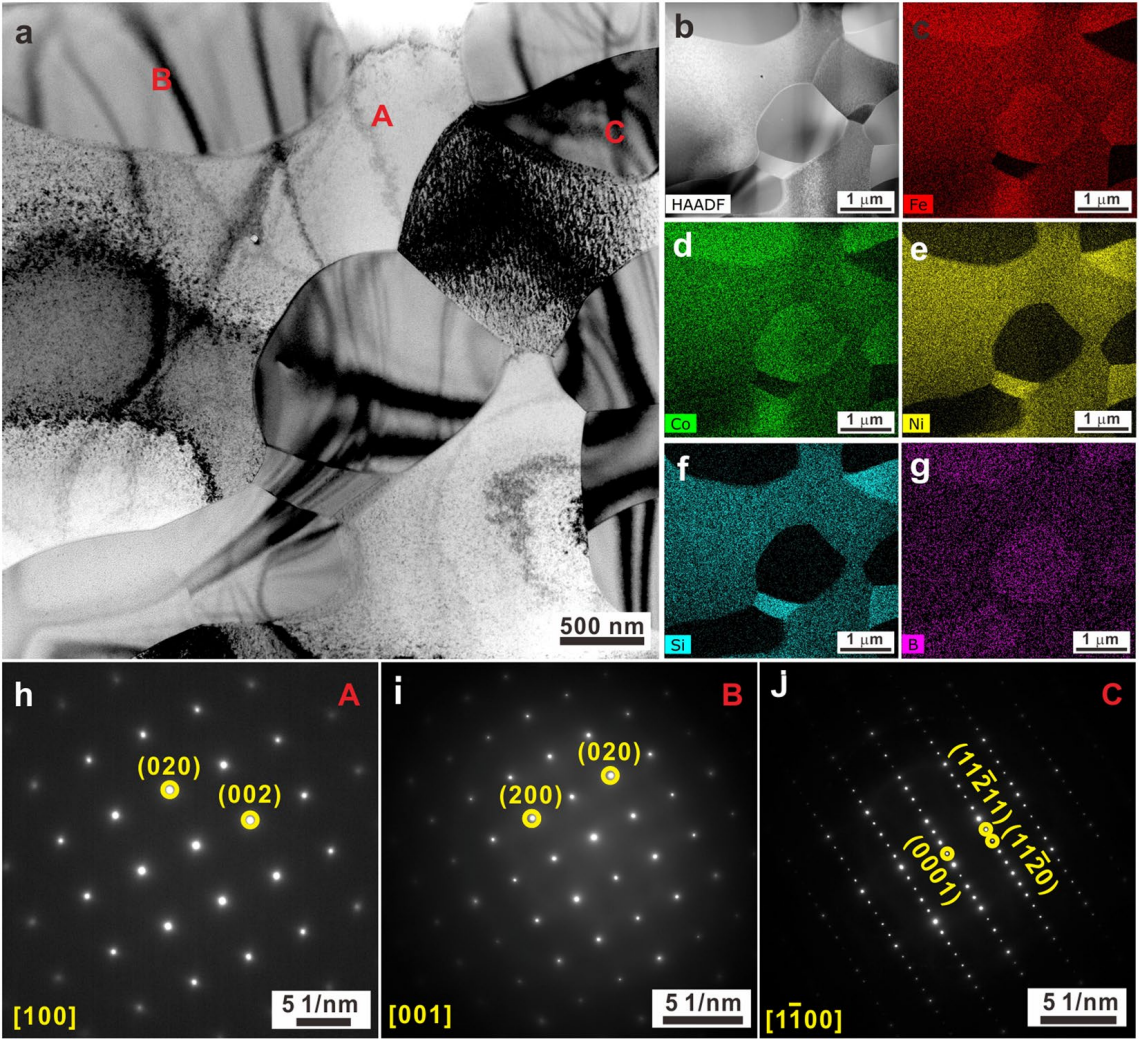
(**a**) Bright-field TEM image and (**b**) HAADF-STEM image of the 1118QC sample as well as its corresponding chemical distributions of elements (**c**) Fe, (**d**) Co, (**e**) Ni, (**f**) Si, and (**g**) B; SAED patterns of the regions (**h**) A, (**i**) B, and (**j**) C in (**a**).

Figure 1a shows the XRD patterns for the as-cast and annealed samples subjected to different cooling methods. All samples are fully crystalline and contain three kinds of crystalline phases. Being different from previous results reported for Fe_26.7_Co_26.7_Ni_26.6_Si_9_B_11_ metallic glass composites^39,40^, not only an *fcc* solid solution phase but also tetragonal crystals (Fe_2_B, Co_2_B or Ni_2_B) as well as a small amount of unknown crystals are observed in all the present samples. The positions of the diffraction peaks of the *fcc* phase remain unchanged with increasing annealing temperature. Even though the samples were annealed at same temperatures but cooled under different cooling rates, no obvious crystalline phase changes can be observed based on the XRD results, implying that no *fcc* to *bcc* transition has occurred during quasi-equilibrium solidification. In order to check microstructural features of the investigated samples, SEM and TEM together with EDX and HAADF-STEM measurements were performed, respectively (Figs 1–5, S1 and S2). Figures 1b and S1 show the microstructural features and chemical distributions of the as-cast Fe_26.7_Co_26.7_Ni_26.7_Si_8.9_B_11_ samples: a typical hypoeutectic structure can be observed, where *fcc* primary dendrites coexist with the eutectic matrix. The eutectic structures (inset in Fig. 1b) in the interdendritic regions display a characteristic lamellar feature. EDX mapping (Fig. S1 in the Supplementary Materials) preliminarily reveals that the primary dendritic phase is rich in Fe, Co, Ni, and Si while the eutectic structures are rich in Fe, Co, and B.

TEM together with HAADF-STEM measurements were conducted on the as-cast samples in order to accurately identify all the existing phases. The bright-field TEM micrograph in Fig. 2a exemplifies both the dendritic crystals and the eutectic structures. Indeed, three different crystalline phases alternately exist inside the eutectic structure, which becomes more obvious in HAADF-STEM images (Fig. 2b). The dendritic crystals (region A in Figs 2a and 3a) mainly consist of Fe, Co, and Ni together with some Si and a small amount of B (Fig. 2b–g), which agrees well with EDX results mentioned above. The selected area electron diffraction (SAED) patterns recorded at the region A in Fig. 3a indicate that the dendritic crystals are the *fcc* phase, whose symmetry space group is Fm-3m (Fig. 3d). Previous investigations have shown that the primary *fcc* phase coexists with a glassy matrix in the case of Fe_26.7_Co_26.7_Ni_26.7_Si_8.9_B_11_ crystalline-amorphous ribbons^40^. Since the applied cooling rate for our bulk samples is relatively lower than that achieved for ribbon samples, no amorphous phase can be induced but a eutectic structure develops together with the primary *fcc* dendrites upon solidification. Besides the precipitation of the *fcc* phase (Fig. 2) within the eutectic structures, another two phases were also detected, which correspond to the regions B and C in Fig. 2a. The region B is rich in Fe, Co, and B while the region C is rich in Ni and Si (Fig. 2b–g). The corresponding SAED pattern (Fig. 3e) which obtains in region B in Figs 2a and 3b, respectively, suggests that the region B corresponds to tetragonal (Fe,Co)_2_B with I4/mcm symmetry space group (Fig. 3e). The region C which exists between the *fcc* and (Fe,Co)_2_B crystals in Figs 2a and 3c is identified as nano-scale hexagonal N_31_Si_12_ (symmetry space group: P321). These findings imply that the hypoeutectic microstructures of the as-cast samples consist of primary *fcc*, eutectic *fcc*, (Fe,Co)_2_B, and a little N_31_Si_12_ phases.

In order to tailor their hypoeutectic microstructures, the as-cast samples were annealed at moderate temperatures (i.e. 786 K or 852 K for 30 min) followed by QC or QC, respectively. Figure 4a–d reveals that the content of the primary *fcc* dendrites increases gradually while parts of the eutectic structures start to decompose into *fcc* and (Fe,Co)_2_B phases together with a small amount of Ni_31_Si_12_ intermetallic compounds for the 786FC, 786QC, 852FC, and 852QC samples. Annealing of the as-cast samples at moderate temperatures occurs under quasi-equilibrium conditions, causing solute redistribution. The induced changes strongly depend on the solid solubility of the constituent elements at different temperatures^41^. In our case, when the melt was quenched into the as-cast HEAs, both the primary *fcc* dendrites and the (Fe,Co)_2_B crystals are supersaturated. Hence when the 786FC, 786QC, 852FC, and 852QC samples are subsequently annealed at moderate temperatures, the eutectic structures tend to coarsen, which is usually linked with the limited solid solubility of the constituents at moderate temperatures^41^. The chemical compositions of the primary *fcc* dendrites and the eutectic phases were roughly determined by EDX and are listed in Table 1. However, it is difficult to accurately detect the content of B due to the limitations of EDX measurements for light element detection and analysis. Therefore, only the contents of Fe, Ni, Co, and Si were measured for the present samples. The chemical compositions of the *fcc* dendrites remain almost constant even though it seems that both the Ni and Si contents slightly increase with increasing annealing temperature. For the (quasi)eutectic structures, both the Fe and Co contents slightly increase while both the Ni and Si contents gradually decrease after low-temperature heat treatments. This indicates that both Ni and Si gradually dissolve into the primary *fcc* dendrites upon low-temperature heat treatments, and simultaneously the eutectic structures coarsen compared to the as-cast samples.

**Table 1:** Average chemical compositions of the *fcc* phase, (quasi) eutectic phases, and (Fe,Co)_2_B intermetallics in different samples, respectively.

Samples	Elements	*fcc* phase	(Quasi) eutectic phase	(Fe,Co)_2_B intermetallics
As-cast	Fe	32.2 ± 2.9	38.0 ± 2.9	—
	Co	29.4 ± 2.8	19.1 ± 2.8	—
	Ni	29.0 ± 3.2	33.8 ± 3.2	—
	Si	9.4 ± 5.2	9.1 ± 5.2	—
786FC	Fe	32.1 ± 2.9	41.3 ± 2.9	—
	Co	29.5 ± 2.8	20.7 ± 2.8	—
	Ni	29.0 ± 3.2	31.0 ± 3.2	—
	Si	9.4 ± 5.2	7.0 ± 5.2	—
786QC	Fe	32.5 ± 2.9	49.4 ± 2.9	—
	Co	29.1 ± 2.8	20.1 ± 2.8	—
	Ni	28.8 ± 3.2	22.8 ± 3.2	—
	Si	9.6 ± 5.2	7.7 ± 5.2	—
852FC	Fe	32.3 ± 2.9	38.3 ± 2.9	—
	Co	29.6 ± 2.8	34.6 ± 2.8	—
	Ni	29.1 ± 3.2	19.7 ± 3.2	—
	Si	9.0 ± 5.2	7.4 ± 5.2	—
852QC	Fe	31.8 ± 2.9	49 ± 2.9	—
	Co	29.4 ± 2.8	21.3 ± 2.8	—
	Ni	29.2 ± 3.2	22.3 ± 3.2	—
	Si	9.6 ± 5.2	7.4 ± 5.2	—
1010FC	Fe	30.6 ± 2.9	—	35.5 ± 2.9
	Co	29.0 ± 2.8	—	34.6 ± 2.8
	Ni	31.1 ± 3.2	—	25.0 ± 3.2
	Si	9.3 ± 5.2	—	4.9 ± 5.2
1010QC	Fe	31.5 ± 2.9	—	34.6 ± 2.9
	Co	29.6 ± 2.8	—	34.2 ± 2.8
	Ni	29.6 ± 3.2	—	24.8 ± 3.2
	Si	9.0 ± 5.2	—	6.4 ± 5.2
1118QC	Fe	29.0 ± 2.9	—	40.0 ± 2.9
	Co	27.7 ± 2.8	—	39.0 ± 2.8
	Ni	32.6 ± 3.2	—	19.2 ± 3.2
	Si	10.7 ± 5.2	—	1.8 ± 5.2
1118FC	Fe	28.8 ± 2.9	—	40.7 ± 2.9
	Co	27.5 ± 2.8	—	39.3 ± 2.8
	Ni	32.6 ± 3.2	—	18.4 ± 3.2
	Si	11.1 ± 5.2	—	1.6 ± 5.2

In order to further confirm the microstructural evolutions, the as-cast samples were annealed at high temperatures (i.e. 1010 K or 1118 K for 30 min) followed by FC or QC, respectively. As shown in Fig. 1a, the crystalline phases remain the same for the 1010FC, 1010QC, 1118FC, and 1118QC samples. However, the SEM images (Fig. 4e–h) of these samples clearly display different microstructural features compared with the as-cast specimens and the samples annealed at moderate temperatures. No distinct eutectic structures can be observed, but rather a quasi-duplex structure develops in the present HEAs: some dark particles are present in the interdendritic regions between the primary dendrites. The distributions of different constituent elements are shown in the EDX maps displayed in Fig. S2 in the Supplementary Materials. Based on the EDX maps, the primary dendrites are also rich in Si and Ni while the dark particles are rich in B, Fe, and Co. In order to further confirm the *fcc* and (Fe,Co)_2_B phases, and especially the Ni_31_Si_12_ intermetallic compounds, TEM and HAADF-STEM investigations were conducted on the 1118QC samples. As shown in Fig. 5a, no obvious eutectic structures can be observed, further confirming the decomposition of the eutectic structures after high-temperature heat treatments and different chemical distributions of the observed crystals compared with the samples annealed at low temperatures. According to HAADF-STEM images (Fig. 5b–g), the region A corresponding to primary dendrites is rich in Fe, Ni, and Co while contains some Si and a small amount of B. The dendrites can be identified as *fcc* crystals based on their SEAD patterns (Fig. 5h). Compared with the samples annealed at low temperatures, the *fcc* crystals coalesce with each other and become the dominant structural framework. Meanwhile, some dark particles can be seen to consist of two different crystals (regions B and C). Being similar to the as-cast samples, region B in Fig. 5a–g is rich in Fe and Co and contains some B. Hence, these crystals within this region can be identified as (Fe,Co)_2_B phase as corroborated by SEAD analysis (Fig. 5i). The region C corresponds to a small volume fraction of nano sale Ni_31_Si_12_ crystals (region C in Fig. 5a–g) which exist adjacent to (Fe,Co)_2_B intermetallic compounds and *fcc* crystals. Furthermore, only the chemical compositions of the primary *fcc* dendrites and (Fe,Co)_2_B intermetallic compounds for the samples annealed at high temperatures were determined by EDX and are listed in Table 1 since it is difficult to detect Ni_31_Si_12_ crystals though EDX measurements in the SEM due to their nanoscale size and limited amount. The contents of Fe and Co in the primary *fcc* dendrites obviously reduce while the contents of Ni and Si gradually increase. Meanwhile, the contents of Fe and Co in the (Fe,Co)_2_B intermetallic compounds seem to increase while the contents of Ni and Si decrease. Since it is difficult to detect Ni_31_Si_12_ crystals though SEM due to their nano sizes and limited amounts, their chemical compositions were measured for the as-cast and the 1010FC samples based on EDX equipped in TEM, which change from about Ni_56.6_Si_11.6_Fe_9.6_Co_22.2_ to Ni_58.6_Si_10.4_Fe_8.6_Co_22.4_, respectively. It is well-known that the solid solubility of constituents in phases usually increases with increasing temperature^41^. Hence, it can be inferred that the eutectic structure should disappear when the annealing temperature is increased.

Furthermore, the results of the annealing experiments described above give no hint for a solid state phase transition from *fcc* to *bcc* phase, as it was observed for high entropy crystalline-amorphous ribbons^39,40^. However, as shown in Fig. S3 in the Supplementary Materials, a *bcc* solid solution indeed precipitates as primary phase during the first crystallization stage of amorphous ribbons and subsequently (Fe,Co)_2_B precipitates form as well. Furthermore, when fully amorphous ribbons are annealed at high temperatures, *fcc* crystals, (Fe,Co)_2_B, and a small amount of Ni_31_Si_12_ intermetallic compounds are found but no *bcc* phase, implying the occurrence of a solid state phase transition from *bcc* to *fcc* phase. Therefore, the *fcc* crystals in a glassy matrix reported by Wei *et al*.^39,40^ for crystalline-amorphous composites should precipitate from the melt during rapid solidification. In the past decades, it has been shown that the primary precipitates forming from melts during (rapid) solidification are usually different from the primary crystallization products from metallic glasses during heating^42–44^. Hence, annealing Fe_26.7_Co_26.7_Ni_26.7_Si_8.9_B_11_ crystalline-amorphous ribbons at a relatively moderate temperature of 788 K causes the stable *fcc* phase in the glassy matrix to transform into the low-temperature metastable *bcc* phase^39,40^ due to different phase stabilities of both solid solutions during non-equilibrium solidification^45–48^. In our case, only the stable *fcc* phase and eutectic structures are retained at room temperature during non-equilibrium solidification, but the stable *fcc* phase does not transform into the metastable *bcc* phase due to the quasi-equilibrium conditions during annealing.

### Mechanical properties of as-cast and annealed HEA samples

**Figure 6:**
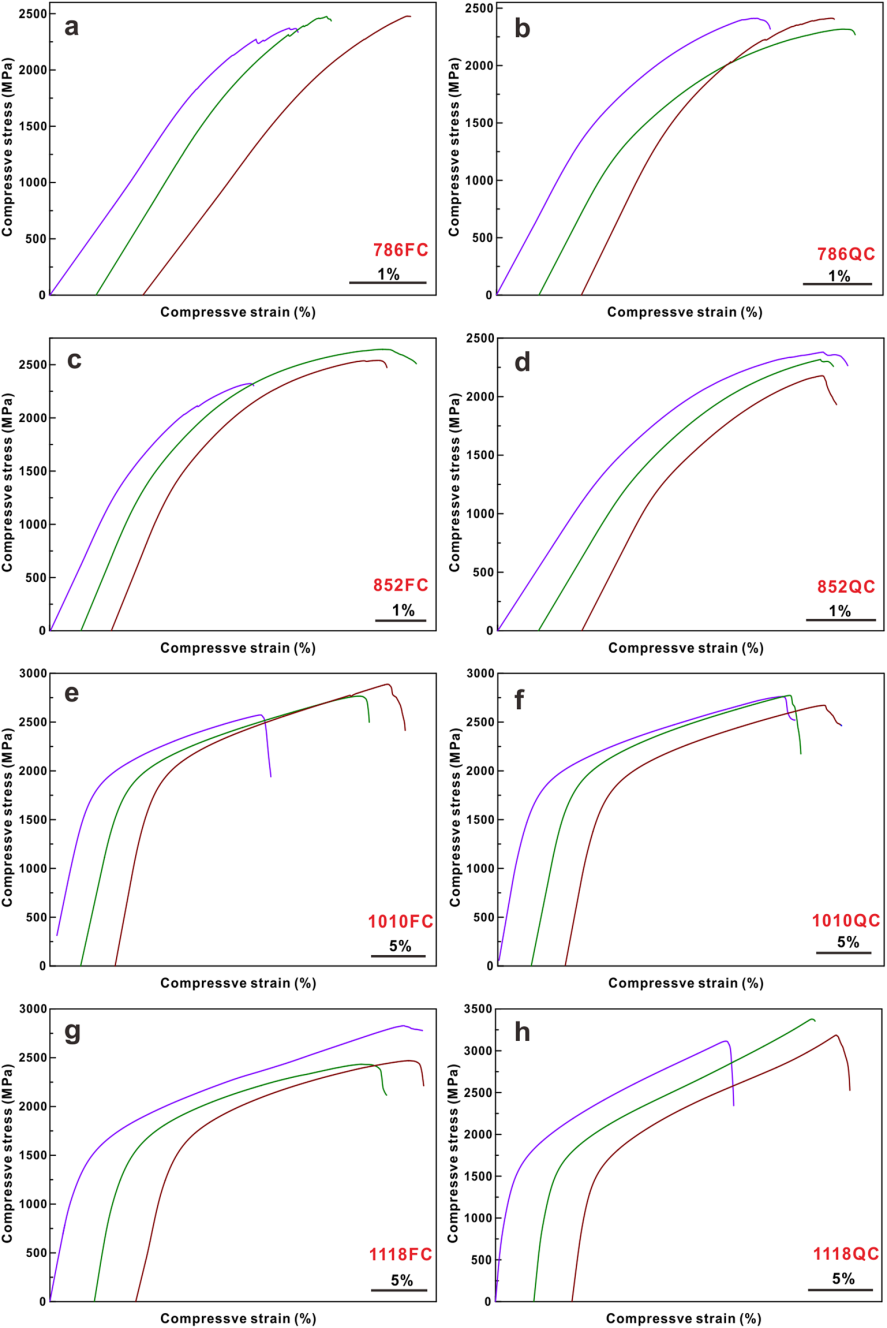
Room temperature engineering stress-strain curves in compression of the (**a**) 786FC, (**b**) 786QC, (**c**) 852FC, (**d**) 852QC, (**e**) 1010FC, (**f**) 1010QC, (**g**) 1118FC, and (**h**) 1118QC samples.

Since different microstructural features can be induced for the samples upon annealing, it is necessary to investigate their mechanical properties. Figure 6 displays the engineering strain-stress curves in compression at room temperature for differently annealed samples. All samples exhibit not only high yield strength but also macroscopic plasticity at room temperature (Fig. 6). Their corresponding characteristic mechanical data is listed in Table 2. The 786FC samples show only a small plastic strain of 0.9 ± 0.1% while the plastic strains of the 796QC, 852FC, and 852QC samples are enhanced to 2.5 ± 0.6%, 3.7 ± 1.4%, and 2.5 ± 0.4%, respectively (Table 2 and Fig. 6a–d). When the annealing is performed at high temperatures (i.e. 1010 K or 1118 K), the plastic strains of the 1010FC, 1010QC, 1118FC, and 1118QC samples at room temperature increase to 20.1 ± 3.7%, 23.0 ± 1.7%, 25.7 ± 4.4%, and 39.8 ± 3.4%, respectively (Table 2 and Fig. 6e–h). The samples annealed at moderate temperatures display relatively larger yield strength and ultimate strength of about 1500 MPa and 2200 MPa, respectively, whereas the samples annealed at high temperatures show lower yield strength and ultimate strength values which are still larger than 900 MPa and 2500 MPa, respectively (Table 2). Hence, it can be concluded that the yield strength gradually decreases while both the ultimate strength and the plastic strain increase with gradually increasing annealing temperature from 786 K to 1118 K. Besides, the QC samples exhibit a slightly lower yield strength and ultimate strength than the furnace cooled specimens when the annealing temperature is below 1118 K. The 1118QC samples show relatively smaller yield strength but a larger ultimate strength (Table 2).

**Table 2:** Average yield strength, ultimate strength, and plastic strains of the samples after different heat treatments.

Samples	yield strength (MPa)	Ultimate strength (MPa)	Plastic strain (%)
786FC	2111 ± 40	2438 ± 61	0.9 ± 0.1
786QC	1595 ± 164	2377 ± 54	2.5 ± 0.6
852FC	1585 ± 53	2500 ± 168	3.7 ± 1.4
852QC	1557 ± 108	2291 ± 103	2.5 ± 0.4
1010FC	1469 ± 58	2739 ± 158	20.7 ± 3.7
1010QC	1378 ± 146	2731 ± 56	23.0 ± 1.7
1118FC	1159 ± 150	2577 ± 222	25. 7 ± 4.4
1118QC	918 ± 78	3227 ± 138	39.8 ± 3.4

**Figure 7:**
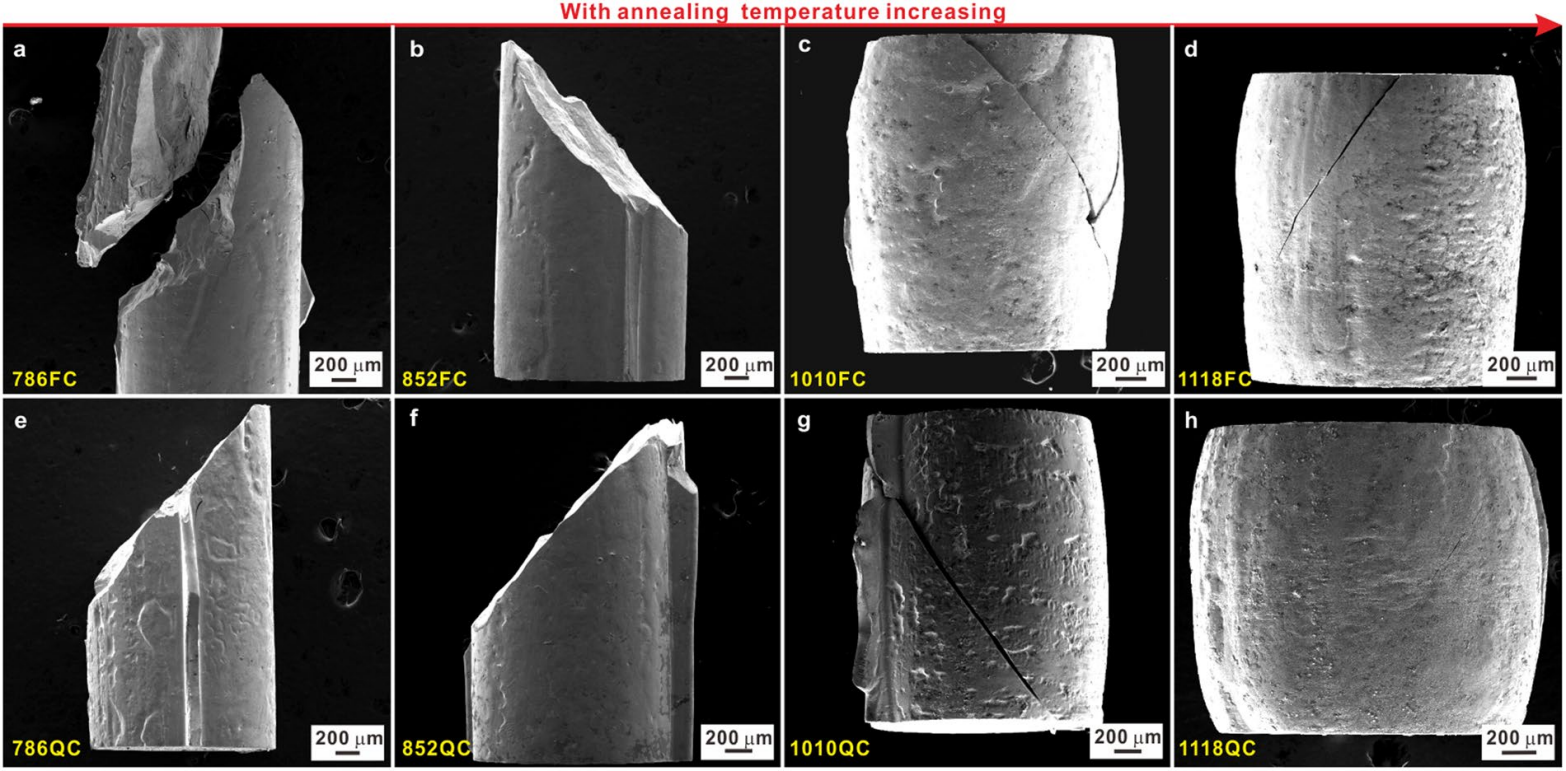
Fracture morphologies of the (**a**) 786FC, (**b**) 852FC, (**c**) 1010FC, (**d**) 1118FC, (**e**) 786QC, (**f**) 852QC, (**g**) 1010QC, and (**h**) 1118QC samples.

**Figure 8:**
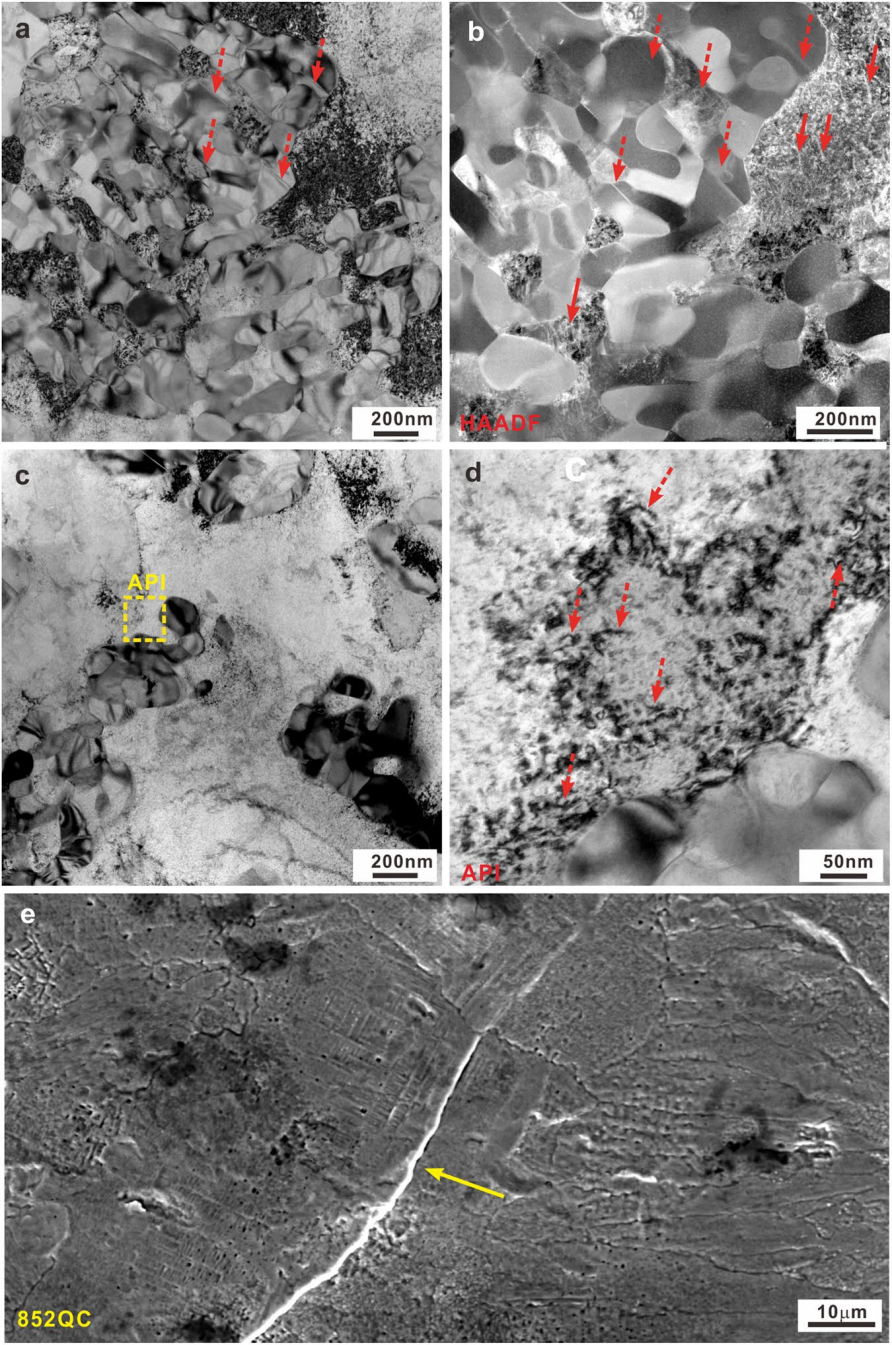
(**a**) TEM image and (**b**) HAADF-STEM image of the eutectic regions, (**c**) Bright-field TEM image of the primary *fcc* regions and (**d**) the corresponding HRTEM image of the API region, and (**e**) SEM image of the lateral fractures surfaces for the 852QC sample.

**Figure 9:**
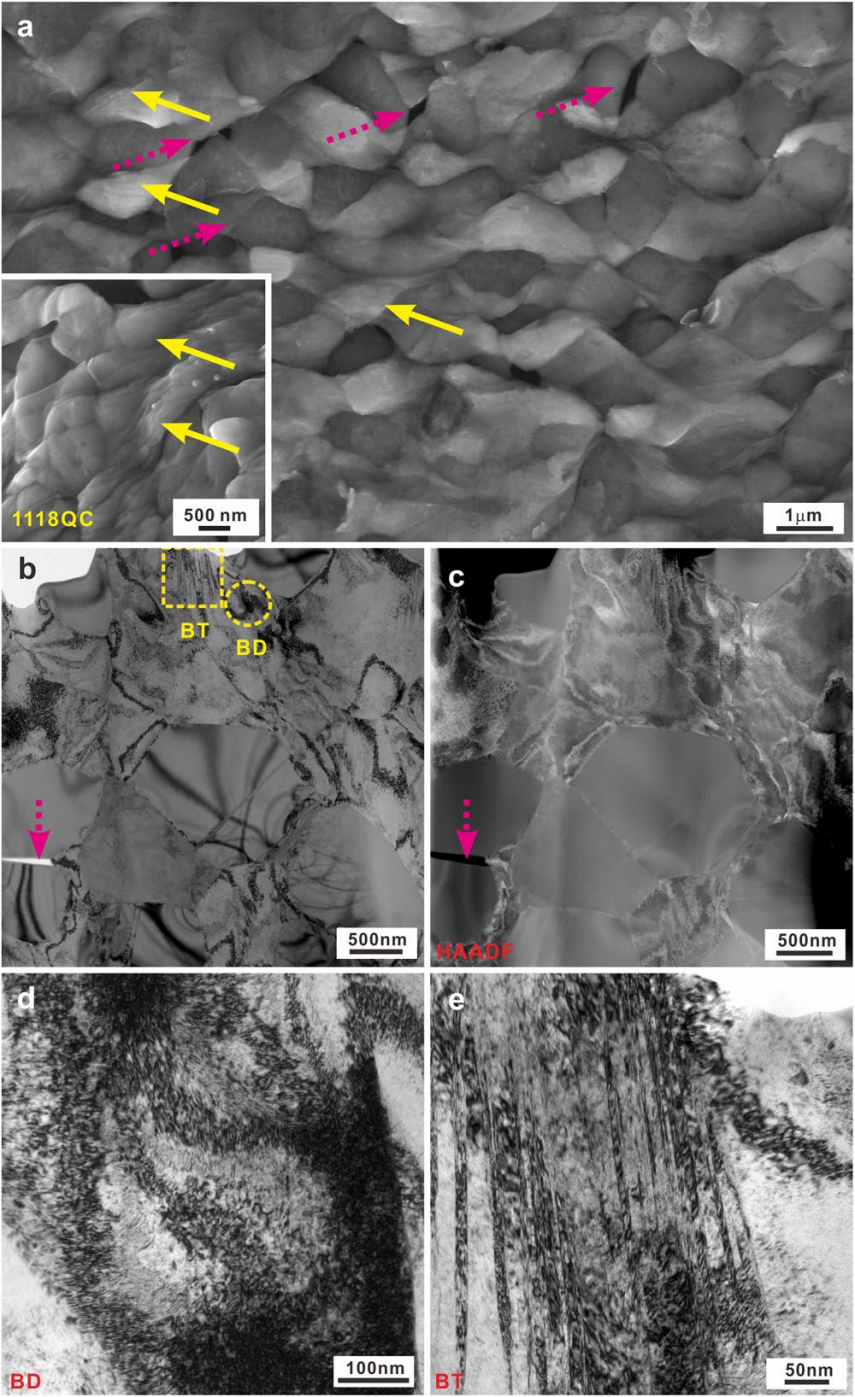
(**a**) SEM image of the lateral fractures surfaces, (**b**) TEM and (**c**) HAADF-STEM images, the corresponding HRTEM images of the (**d**) BD and (**e**) BT regions for the 1118QC sample.

**Figure 10:**
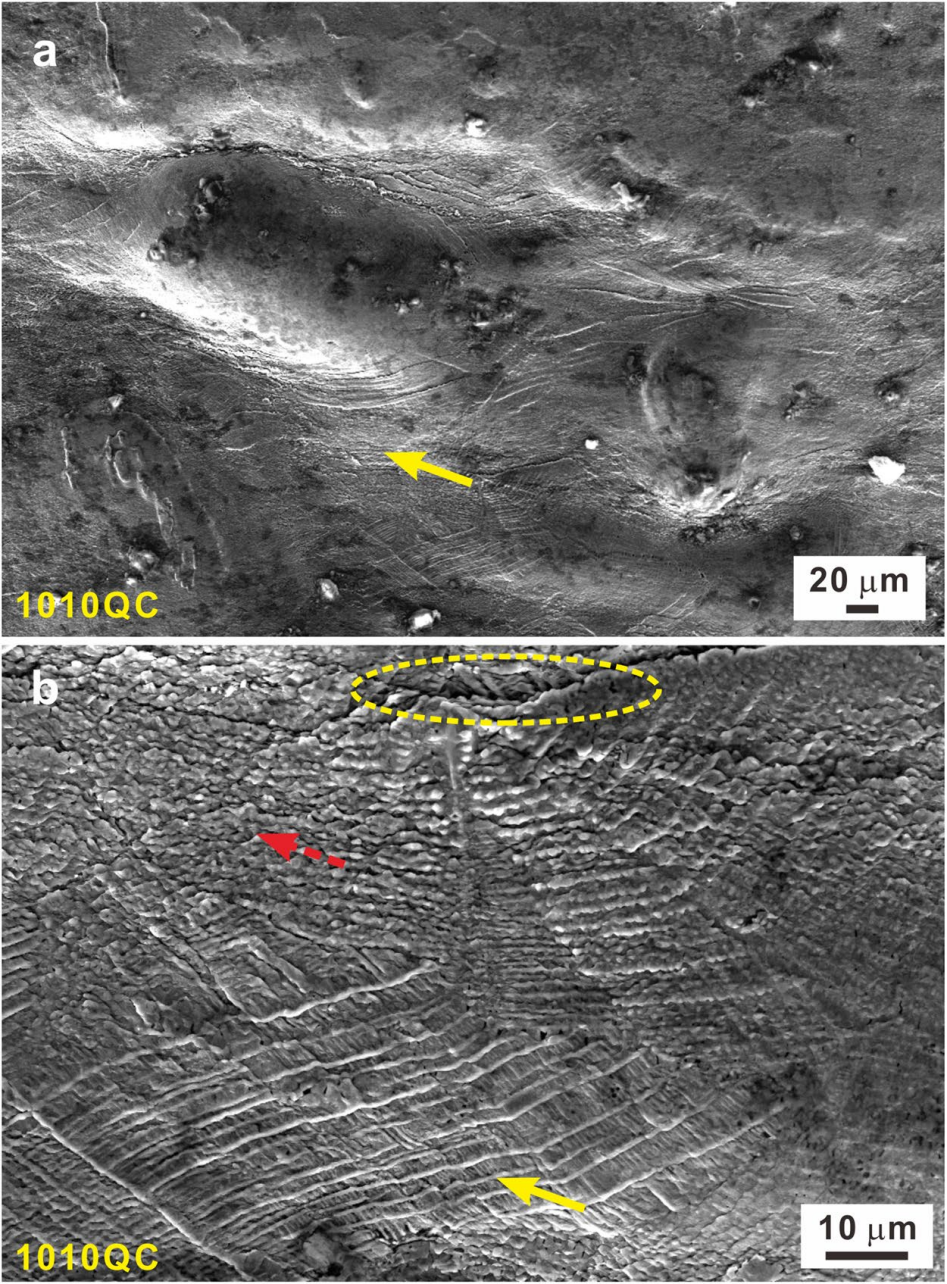
(**a**) Lateral fractures surfaces and (**b**) their local enlarged image for the 1010QC samples.

In order to clarify the deformation mechanism responsible for the transition from a brittle to ductile type deformation at room temperature for the investigated samples, we evaluated the morphologies of the compressive lateral and fracture surfaces (Figs 7–10 and S4) by taking the above mentioned microstructural features into account (Figs 2–5). Figure 7a–h and S4 reveal that the samples after fracture indeed show a transition from a brittle to a ductile fracture mode with increasing annealing temperature. When the annealing temperature is below 1010 K, the samples fail in a shear mode under compression and the fracture angle is 43° ± 0.5°. However, the samples annealed at 1118 K do not fracture completely and show a drum-like shape, further confirming their intrinsic room-temperature ductility. When the annealing temperature is between 852 K and 1118 K, i.e. 1010 K in our case, both characteristic fracture features can be observed. In attempt to reveal the change of the dominant deformation mechanism for the investigated samples, the lateral surfaces and microstructures of the 852QC, 1010QC, and 1118QC samples were checked. Since the microstructures of the 852QC samples are composed of primary *fcc* phase, secondary-precipitated phases and remaining eutectic structures, the remaining eutectic structures still keep an effective structural framework while the isolated *fcc* crystals do not impinge with each other (Fig. 4d). TEM and HAADF-STEM measurements (Fig. 8a–b) also confirm such microstructural features for the 852QC sample and unveil the formation of a large amount of micro cracks can be induced within the intermetallic compounds insides the eutectic structures (see the dotted arrows). Within the *fcc* crystals neighboring eutectic regions, a few discontinuous slip bands (see the solid arrows in Fig. 8b) also can be observed. Besides, as shown in Fig. 8b–d, a lot of dislocations can be observed within the *fcc* crystals neighboring eutectic regions and within the *fcc* crystals inside eutectic regions, respectively. However, within other *fcc* regions away from eutectic regions, it is difficult to observe the formation of slip bands and dislocation (Fig. 8d), implying that the plastic deformation originating from the dislocation multiplication (see the dotted arrows in Fig. 8d) mainly concentrates around the regions around and within eutectic structures, respectively. Therefore, the contribution of the plastic deformation from the limited amount of *fcc* crystals during deformation causes that the eutectic structural framework cannot bear relatively large plastic deformation, leading to the formation of a large amount of micro-cracks (Fig. 8e) around the interfaces between the *fcc* crystals and eutectic regions and within eutectic regions, respectively.

However, for the 1118QC samples, the isolated *fcc* crystals become the effective structural framework due to their rather large volume fraction and isolated (Fe,Co)_2_B intermetallic compounds appears due to the complete decomposition of the eutectic structure (Fig. 4h). During deformation, the plastic deformation is concentrated within the *fcc* crystals (see the solid arrow in Fig. 9a) and the isolated (Fe,Co)_2_B intermetallic compounds act as reinforcing particles. Therefore, for the 1118QC samples after deformation, the TEM and HAADF images show that the plastic deformation mainly occurs within all the *fcc* crystals neighboring and away from the intermetallic particles (Fig. 9b,c) but not the intermetallic compounds. As shown in Fig. 9 d,e, a high density of dislocations can be observed accompanied with a few nano twins, which can provide efficient plastic strains during deformation and induce the formation of multiple and fine shear bands (Fig. 9a). When the (Fe,Co)_2_B intermetallics reach their ultimate strength, some cracks appear and gradually link with each other (see the dotted arrows in Fig. 9a–c), resulting in failure of the samples. Furthermore, for the 1010QC samples, both the *fcc* and (Fe,Co)_2_B crystals are not fully isolated but still connected with each other (Fig. 4g).Then the competition between the formation of cracks and dislocations results in the formation of a large amount of shear bands during deformation (Fig. 10a) and both deformation features (see the dotted and solid arrows in Fig. 10b), leading to the achievement of both relatively large yield strength and plastic strain (Table 2 and Fig. 6f). Until the formation of large cracks (see the circle in Fig. 10b), the 1010QC sample starts to fail.

## Conclusions

In this work, the correlation between microstructural features and mechanical properties of Fe_26.7_Co_26.7_Ni_26.7_Si_8.9_B_11_ high entropy alloys was investigated. By using rapid solidification, primary *fcc* crystals and eutectic structures were formed in as-cast samples. The eutectic structures consist of *fcc*, (Fe,Co)_2_B and a small amount of Ni_31_Si_12_ crystalline phases. When the as-cast samples are annealed at moderate temperatures (i.e. 786 or 852 K) followed by furnace cooling or quenching, respectively, the eutectic structures become coarsened and parts of them start to decompose into *fcc* solid solution, (Fe,Co)_2_B, and some nano-scale Ni_31_Si_12_ intermetallic compounds, leading to an increasing volume fraction of *fcc* phase. When the annealing temperature increases to 1010 K, no obvious eutectic structures can be observed. Meanwhile, both *fcc* solid solution crystals and (Fe,Co)_2_B intermetallic compounds impinge with each other forming a structural framework, while the amount of *fcc* dendrites is larger than the volume fraction of (Fe,Co)_2_B intermetallics. When the annealing temperature is further increased to 1118 K, the *fcc* solid solution becomes the dominant structural framework while the isolated (Fe,Co)_2_B intermetallic compounds can be treated as the dominant reinforcing particles distributed in the interdendritic regions. Due to the gradual microstructural transition from hypoeutectic to quasi-duplex structures, the transition from a brittle to ductile type deformation behaviors can be observed. Hence, the mechanical properties of the investigated HEAs can be adjusted by different heat treatments. All specimens exhibit not only high yield strength but also macroscopic plasticity at room temperature. The samples annealed at moderate temperatures (i.e. 786 K or 852 K) show relatively small plastic strain and ultimate strength but high yield strength. When the annealing temperature is increased to high temperatures (i.e. 1010 K or 1118 K), both the plastic strain and the ultimate strength of the annealed samples increase while the yield strength continues to decrease.

## Methods

Ingots with a nominal composition of Fe_26.7_Co_26.7_Ni_26.7_Si_8.9_B_11_ were fabricated by arc-melting appropriate amounts of constituting elements (Fe, Co, Ni, and Si, >99.9% purity) and Fe_45.32_B_54.68_ master alloys (>99.9% purity) under Ti-gettered argon atmosphere. In order to guarantee chemical homogeneity, the master alloys were remelted at least four times before suction casting. During rapid solidification, rods with a diameter of 1.5 mm were prepared using a custom-made suction-casting device under argon atmosphere. Thermal analysis was conducted by differential scanning calorimetry (DSC, METTLER TOLEDO TGA/DSC 1) at a heating rate of 20 K/min. As shown in Fig. S5, the onset and final temperatures (i.e. *T*_*m*_ and *T*_*L*_) of the melting events of the present samples were determined to be 1263 ± 2 K and 1341 ± 2 K, respectively. In order to tailor their microstructures, the as-cast rods were sealed in quartz tubes under an argon atmosphere and then heated to 786 K, 852 K, 1010 K, and 1118 K for 30 min, respectively. Two different procedures were adopted for subsequent cooling: furnace cooling (FC) and quenching (QC) into water. In the following sections, the obtained samples will be denoted based on the annealing temperatures and cooling methods (Table 1). The phase analysis of the as-cast specimens was carried out by X-ray diffraction (XRD, Rigaku D/max-rB) in reflection geometry, scanning electron microscopy (SEM, Gemini 1530) equipped with an energy dispersive X-ray spectroscopy (EDX), and transmission electron microscopy (TEM, JEOL-2100). The samples for the TEM measurements were prepared by a dual focused ion beam system (FIB, HELIOS NanoLab 600i) which was set up in a scanning electron microscopy (SEM, FEI Sirion). The chemical compositions were also double checked by high-angle annular dark-field scanning transmission electron microscopy (HAADF-STEM, TECNAI G2 F30). Room-temperature compression tests were performed on specimens with a height-to-diameter ratio of about 2:1 using an electronic universal testing machine (New SANS, MTS) at an initial strain rate of 2.5 × 10^−4^ s^−1^. The surface morphology of the samples after deformation was investigated by SEM (Gemini 1530).

## Electronic supplementary material

Supplementary materials

## Data Availability

All data and materials involved in this study are included in this published article and its Supplementary File.
