# Supplementary material for: Optimizing mechanical properties of Fe26.7Co26.7Ni26.7Si8.9B11 high entropy alloy by inducing hypoeutectic to quasi-duplex microstructural transition
Source: Sci Rep. 2019 Jan 23;9:360. doi: 10.1038/s41598-018-36464-3 (PMC6344508; doi:10.1038/s41598-018-36464-3)
Supplement: Supplementary file 1 — Supplementary materials [file 41598_2018_36464_MOESM1_ESM.docx]

**Optimizing mechanical properties of Fe_26.7_Co_26.7_Ni_26.7_Si_8.9_B_11_ high entropy alloy by inducing hypoeutectic to quasi-duplex microstructural transition**

Ze-Qun Zhang1, Kai-Kai Song^1,†^, Shu Guo^2^, Qi-Sen Xue^1^, Hui Xing^3^, Chong-De Cao^3^, Fu-Ping Dai^3^, Bernhard Völker^4,5,6^, Anton Hohenwarter^7^, Tapabrata Maity^7^, Niraj Chawake^4^, Jeong-Tae Kim^4^, Li Wang^1^, Ivan Kaban^8^, and Jürgen Eckert^4, 7^

School of Mechanical, Electrical & Information Engineering, Shandong University (Weihai), 264209 Weihai, China. ^2^School of Materials Science and Engineering, Harbin Institute of Technology, 150001 Harbin, China. ^3^Department of Physics, School of Science, Northwestern Polytechnical University, 710072 Xi’an, China. ^4^Erich Schmid Institute of Materials Science, Austrian Academy of Sciences, A-8700 Leoben, Austria. ^5^Materials Chemistry, RWTH-Aachen, D-52074 Aachen, Germany. ^6^Max-Plank-Institut für Eisenforschung GmbH, D-40237 Düsseldorf, Germany. ^7^Department Materials Physics, Montanuniversität Leoben, A-8700 Leoben, Austria. ^8^IFW Dresden, Institute for Complex Materials, D-01069 Dresden, Germany. ^†^ Correspondence and requests for materials should be addressed to K.S. (email: songkaikai8297@gmail.com)

Supplementary Information including:

1. **EXPERIMENTAL PROCEDURE FOR RIBBONS**
2. **FIGURE S1, FIGURE S2, FIGURE S3, FIGURE S4, and FIGURE S5.**

**EXPERIMENTAL PROCEDURE**

Master alloys with a nominal composition of Fe_26.7_Co_26.7_Ni_26.7_Si_8.9_B_11_ were fabricated by arc-melting appropriate amounts of constituting elements (> 99.9% purity) under Ti-gettered argon atmosphere. In order to guarantee chemical homogeneity, the master alloys were remelted at least four times before suction casting. Then the master alloys were remelted using a high frequency furnace under an argon atmosphere and quenched into ribbons with a thickness of 35 ± 5 μm by melt-spinning. The ribbons were annealed in a differential scanning calorimeter (DSC, METTLER TOLEDO TGA/DSC 1) under a flow of purified argon at a heating and cooling rate of 20 K/min. The phase analysis of the as-quenched and the as-annealed ribbons was carried out by X-ray diffraction (XRD, Rigaku D/max-rB) in reflection geometry.


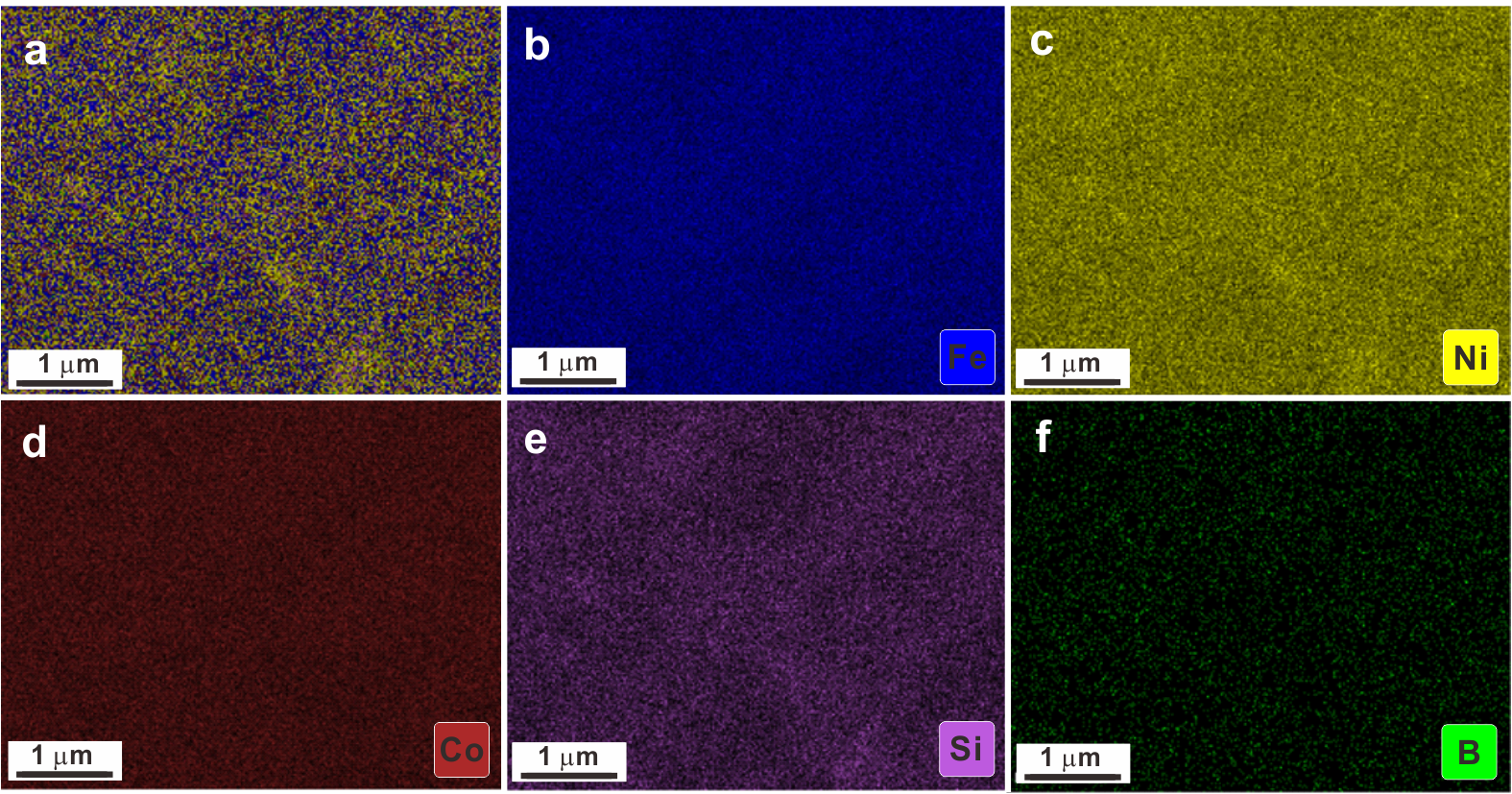


**Figure S1** (**a**) EDX maps of an as-cast sample and the corresponding distributions of elements (**b**) Fe, (**c**) Ni, (**d**) Co, (**e**) Si, and (**f**) B, respectively.


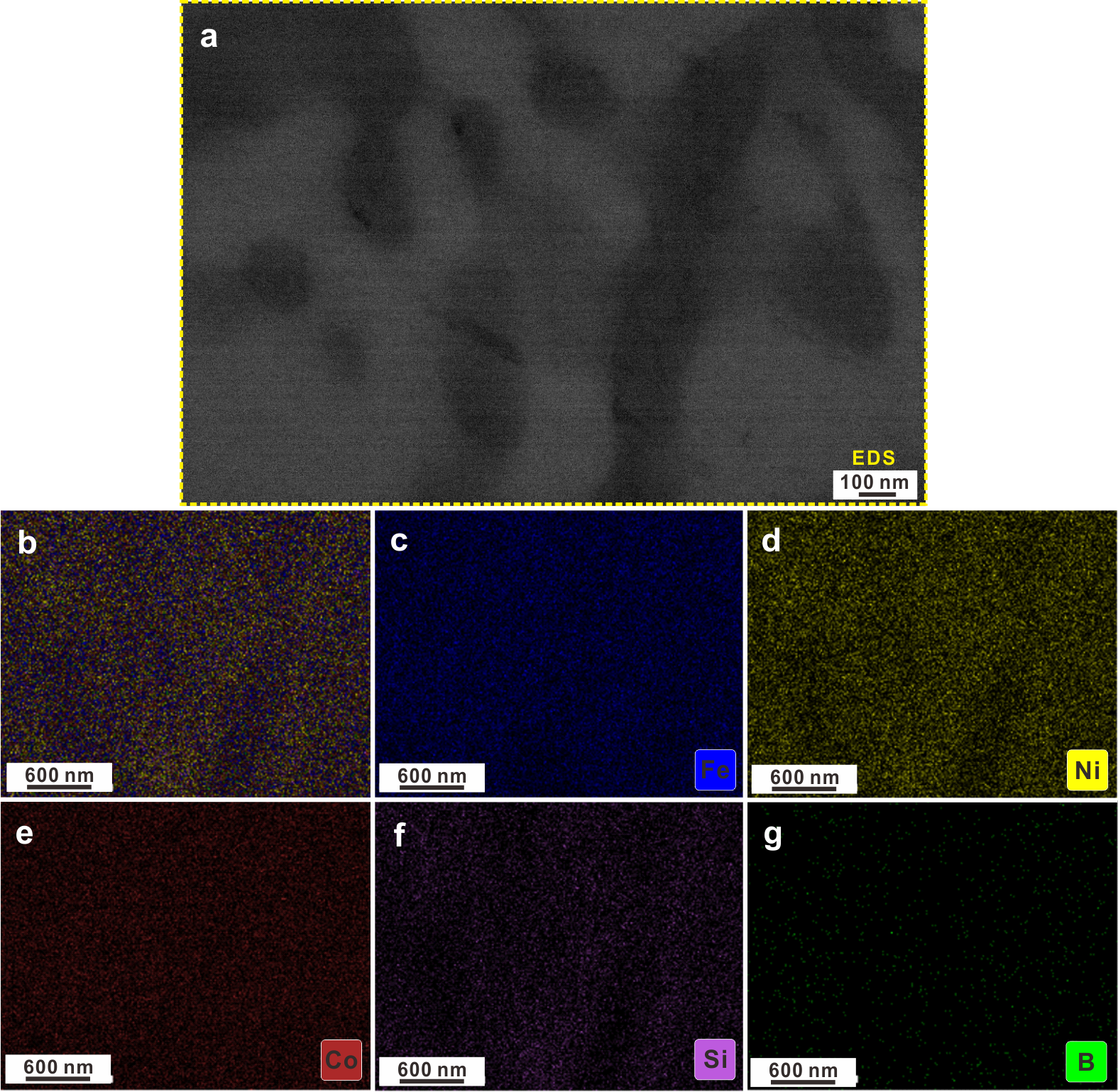


**Figure S2** (**a**) Local magnified SEM image of the 1010FC sample and corresponding (**b**-**g**) EDX maps.


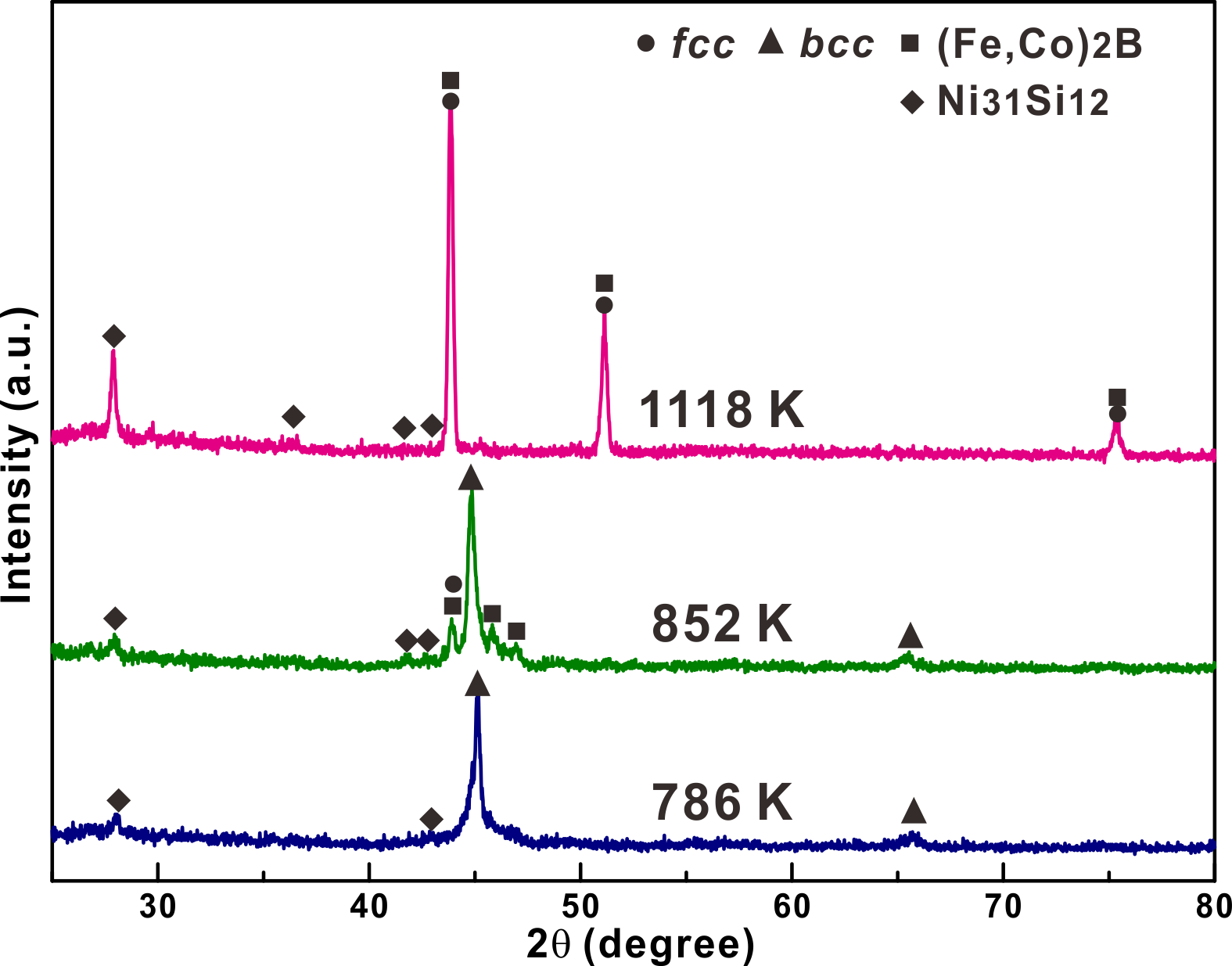


**Figure S3** XRD patterns for as-quenched ribbons annealed at temperatures of 786 K, 852 K, and 1118 K, respectively.


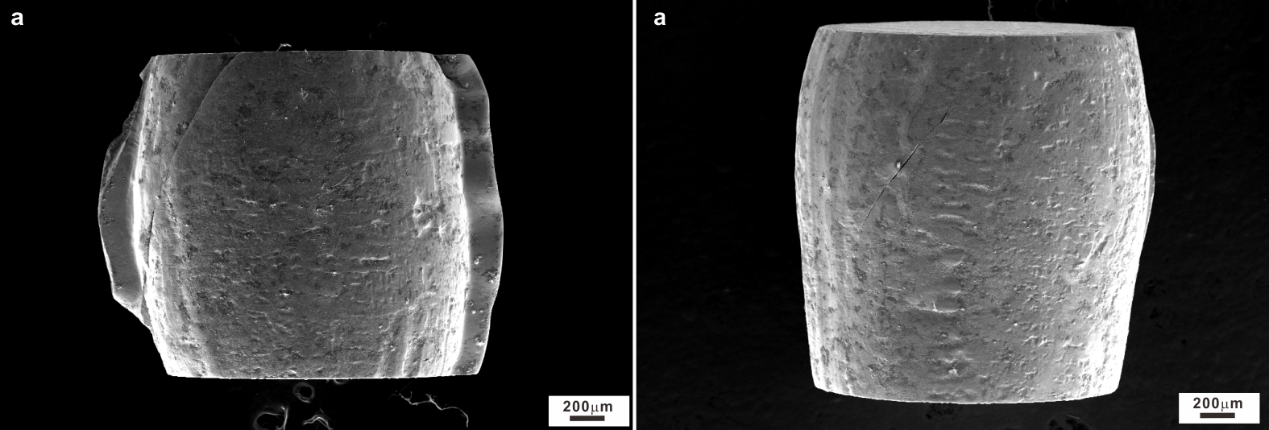


**Figure S4** Lateral fractures surfaces of the 1118QC samples.


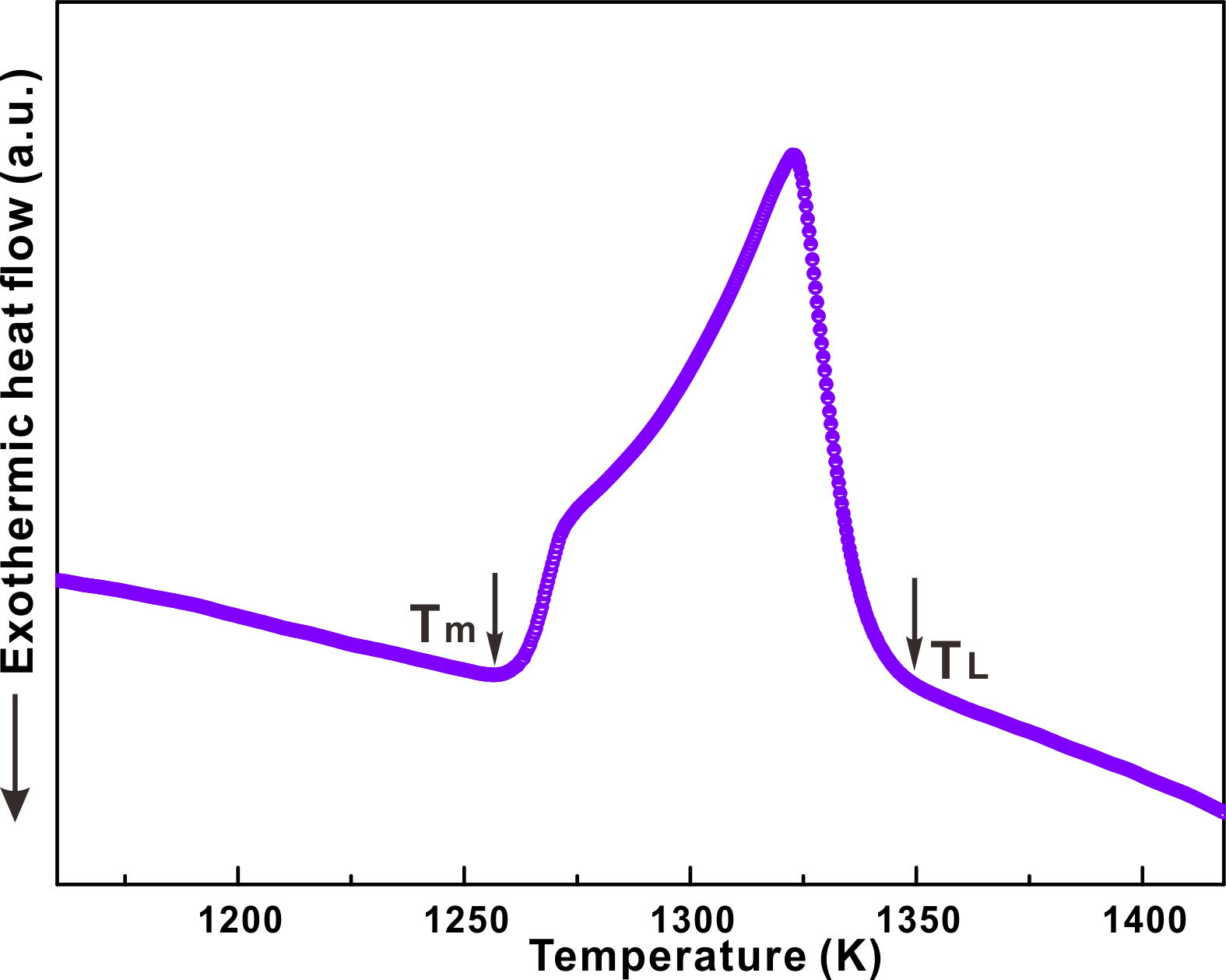


**Figure S5** High-temperature DSC curve (heating rate 20 K/min) of the as-cast sample.
